# Leveraging the Variability of Pharmacovigilance Disproportionality Analyses to Improve Signal Detection Performances

**DOI:** 10.3389/fphar.2021.668765

**Published:** 2021-05-28

**Authors:** Charles Khouri, Thuy Nguyen, Bruno Revol, Marion Lepelley, Antoine Pariente, Matthieu Roustit, Jean-Luc Cracowski

**Affiliations:** ^1^ Pharmacovigilance Unit, Grenoble Alpes University Hospital, Grenoble, France; ^2^ Clinical Pharmacology Department INSERM CIC 1406, Grenoble Alpes University Hospital, Grenoble, France; ^3^ Hypoxia and PhysioPathology, UMR 1300, INSERM, University Grenoble Alpes, Grenoble, France; ^4^ INSERM U1219, Bordeaux Population Health, Team Pharmacoepidemiology, University of Bordeaux, Bordeaux, France; ^5^ Service de Pharmacologie Médicale, Pôle de Santé Publique, CHU de Bordeaux, Bordeaux, France

**Keywords:** pharmacovigilance, disproportionality analyses, signal detection, drug safety, Transparency

## Abstract

**Background:** A plethora of methods and models of disproportionality analyses for safety surveillance have been developed to date without consensus nor a gold standard, leading to methodological heterogeneity and substantial variability in results. We hypothesized that this variability is inversely correlated to the robustness of a signal of disproportionate reporting (SDR) and could be used to improve signal detection performances.

**Methods:** We used a validated reference set containing 399 true and false drug-event pairs and performed, with a frequentist and a Bayesian disproportionality method, seven types of analyses (model) for which the results were very unlikely to be related to actual differences in absolute risks of ADR. We calculated sensitivity, specificity and plotted ROC curves for each model. We then evaluated the predictive capacities of all models and assessed the impact of combining such models with the number of positive SDR for a given drug-event pair through binomial regression models.

**Results:** We found considerable variability in disproportionality analysis results, both positive and negative SDR could be generated for 60% of all drug-event pairs depending on the model used whatever their truthfulness. Furthermore, using the number of positive SDR for a given drug-event pair largely improved the signal detection performances of all models.

**Conclusion:** We therefore advocate for the pre-registration of protocols and the presentation of a set of secondary and sensitivity analyses instead of a unique result to avoid selective outcome reporting and because variability in the results may reflect the likelihood of a signal being a true adverse drug reaction.

## Introduction

Spontaneous reports are a valuable data source to complete the pre-marketing safety profile of medical products, notably for rare or long latency adverse drug reactions (ADRs) (Hartmann et al., 1999; Hauben and Bate, 2009). With the massive increase in new reports received each year by pharmacovigilance centers around the world (e.g. more than 2,700,000 new reports were recorded in the WHO’s pharmacovigilance database in 2019), traditional approaches such as manual review needed to be complemented. Automated screening tools using quantitative methods have thus been developed to detect signals of disproportionate reporting (SDR), i.e. a higher proportions of reporting of ADRs for a studied drug as compared to the other drugs in the database. They allow broad screening to trigger further investigations, to detect more complex dependencies and to prioritize potential signals (Bate and Evans, 2009; Hauben and Aronson, 2009).

These methods include frequentist, Bayesian and machine learning approaches (Hauben and Bate, 2009; Harpaz et al., 2012). In addition, several models (subgroup, stratification or adjustments) can be used to overcome the multitude of biases related to spontaneous reporting rates of ADR, such as media alerts, selective reporting according to ADR severity, or time since the drug was first marketed (Seabroke et al., 2016; Wisniewski et al., 2016; Sandberg et al., 2020). They also permit to overcome disparities in drug usage and pharmacovigilance systems, or to account for risk factors of developing an ADR (e.g. sex, age, underlying conditions) (Raschi et al., 2018; Sandberg et al., 2020). Several studies have assessed and compared the performances of such methods and models, which did not reveal significant differences for signal detection (Harpaz et al., 2013; Candore et al., 2015; Pham et al., 2019). As a result, no consensus exists to date on the best analyses and no gold standard has been defined (Wisniewski et al., 2016). In this context, a large heterogeneity exists in the modalities retained for signal detection from spontaneous reporting, especially regarding the complementary analyses that can be performed to explore the robustness of the detected statistical signals. The variety of the methodological choices that are made may lead to substantial variability in results and, when these appear conflicting in the literature, lead to increase the complexity of their interpretation (Khouri et al., 2021). In this context, we hypothesized that performing a set of standardized analyses relying on different techniques could help appraising the robustness of a signal.

## Methods

In this study, we used the Observational medical outcomes partnership (OMOP) gold standard reference set to assess the diagnostic performances of a set of seven models of two widely used frequentist (Reporting Odds Ratio) and bayesian (Bayesian confidence propagation neural network) disproportionality methods applied to the WHO pharmacovigilance database, Vigibase®.

### Reference Set

The OMOP reference set have been established to facilitate methodological research in drug safety and to allow comparison of signal detection performances of disproportionality analyses. The gold standard consists of 165 true and 234 false drug-event pairs originating from a systematic literature review and natural language processing of structured product labels (Ryan et al., 2013). The reference set spans 181 unique drugs covering antibiotics, nonsteroidal anti-inflammatory drugs, antidepressants, antihypertensives, antiepileptics and glucose lowering drugs. The specific outcomes (acute renal injury, myocardial infarction, acute liver injury, gastrointestinal bleeding) have been selected because they are considered as high priority events in pharmacovigilance for different reasons (Ryan et al., 2013). Acute myocardial infarction and upper gastrointestinal bleeding possess high background rates in the general population, with a different proportion of iatrogenic etiologies identified. Acute kidney and liver injury are important outcomes for post-market drug surveillance as they are the main pathways for drug metabolism and elimination, and because patients with pre-existing conditions are often excluded from phase 3 clinical trials (Trifirò et al., 2009).

### Data Source

All data used for disproportionality analyses were extracted from the WHO pharmacovigilance database, VigiBase, from January 1, 1968 to December 31, 2019. Gathering reports from more than 130 member countries, Vigibase is the largest pharmacovigilance database containing more than 21 million individual case safety reports (ICSRs) submitted by pharmaceutical manufacturers, health professionals, or consumers through national pharmacovigilance systems (Lindquist, 2008).

We identified the four outcomes in Vigibase by using a collection of MedDRA Preferred Terms (PT) or standardized MedDRA queries (SMQ) to match the broader definitions used in the reference set (Supplementary Table S1). (Reich et al., 2013; Ryan et al., 2013)

### Signal Generation

Two disproportionality methods were used in this study, the Reporting Odd Ratio (ROR) used by the European Medicines Agency, and the Bayesian confidence propagation neural network, used by the Uppsala Monitoring Center on behalf of the WHO. A SDR was considered significant if the lower boundary of the 95% confidence interval of ROR (ROR_LB_) was ≥1 and the number of observed drug-event combinations ≥3; or if the lower boundary of the IC 95% confidence interval (IC_LB_) was >0 (Bate et al., 1998; European Medicines Agency and EudraVigilance Expert Working Group, 2006. Guideline on the use of statistical signal detection methods in the EudraVigilance data analysis system. Available on: https://www.ema.europa.eu/en/documents/regulatory-procedural-guideline/draft-guideline-use-statistical-signal-detection-methods-eudravigilance-data-analysis-system_en.pdf, 2006).

We used seven disproportionality models for which the results were very unlikely to be influenced by actual differences in absolute risks of ADR: Model 1: only suspect reports included; Model 2: to assess the influence of pharmacovigilance systems between countries we restricted reports to a specific country (United States); Model 3: restricting reports to those submitted by health care professionals only (physicians or pharmacists); Model 4: restricting the database to the drug’s corresponding therapeutic area (ATC code level 3) to account for difference non-cases populations; Model 5: including only serious cases; Model 6: included only cases reported within 5 years after the drug’s marketing approval date to account for reporting variability according drug time on the market; and Model 7: included suspected and concomitant drugs.

### Evaluation

The performances of the disproportionality models were evaluated through sensitivity, specificity and area under the curve (AUC). ROC curves were plotted for each model. To understand the contribution of the number of positive SDRs alongside the disproportionality values we built logistic regression models both with and without inclusion of the number of positive SDR. In addition, we plotted the predictive capacities (marginal means) of the models according to the number of positive SDR. We postulated that the number of drug-event pairs could impact the disproportionality results; we thus included this variable in the logistic regression models.

Lastly, we calculated and compared median ROR_LB_ and IC_LB_ values, and the median number of positive SDR between true and false drug-event pairs through Mann-Whitney-Wilcoxon tests. A two-sided *p* value < 0.05 was considered significant.

Statistical analyses were performed with R (version 3.6.1). The protocol, data and R codes underlying this article could be found on Open Science Framework (osf.io/a7j3z/)

## Results

### Data and Signal Generation Results

**FIGURE 1:**
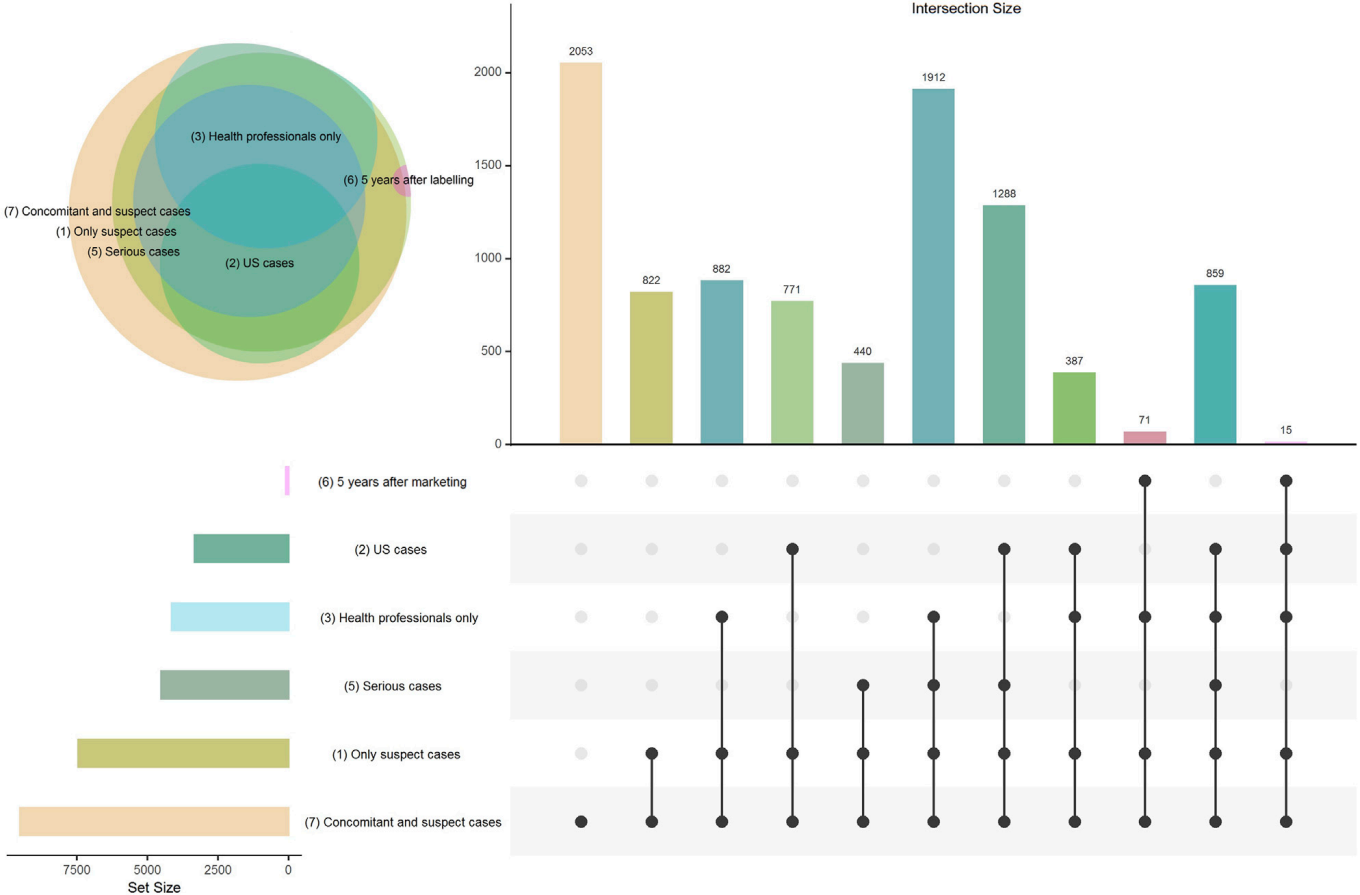
Venn diagram and Upset plot presenting the distribution of cases included in each disproportionality analysis and the overlap between each of the models. Model 4 (subgroups by therapeutic area) is not displayed in the figure because only the comparators were modified in this model (cases correspond to all suspect cases).

**FIGURE 2:**
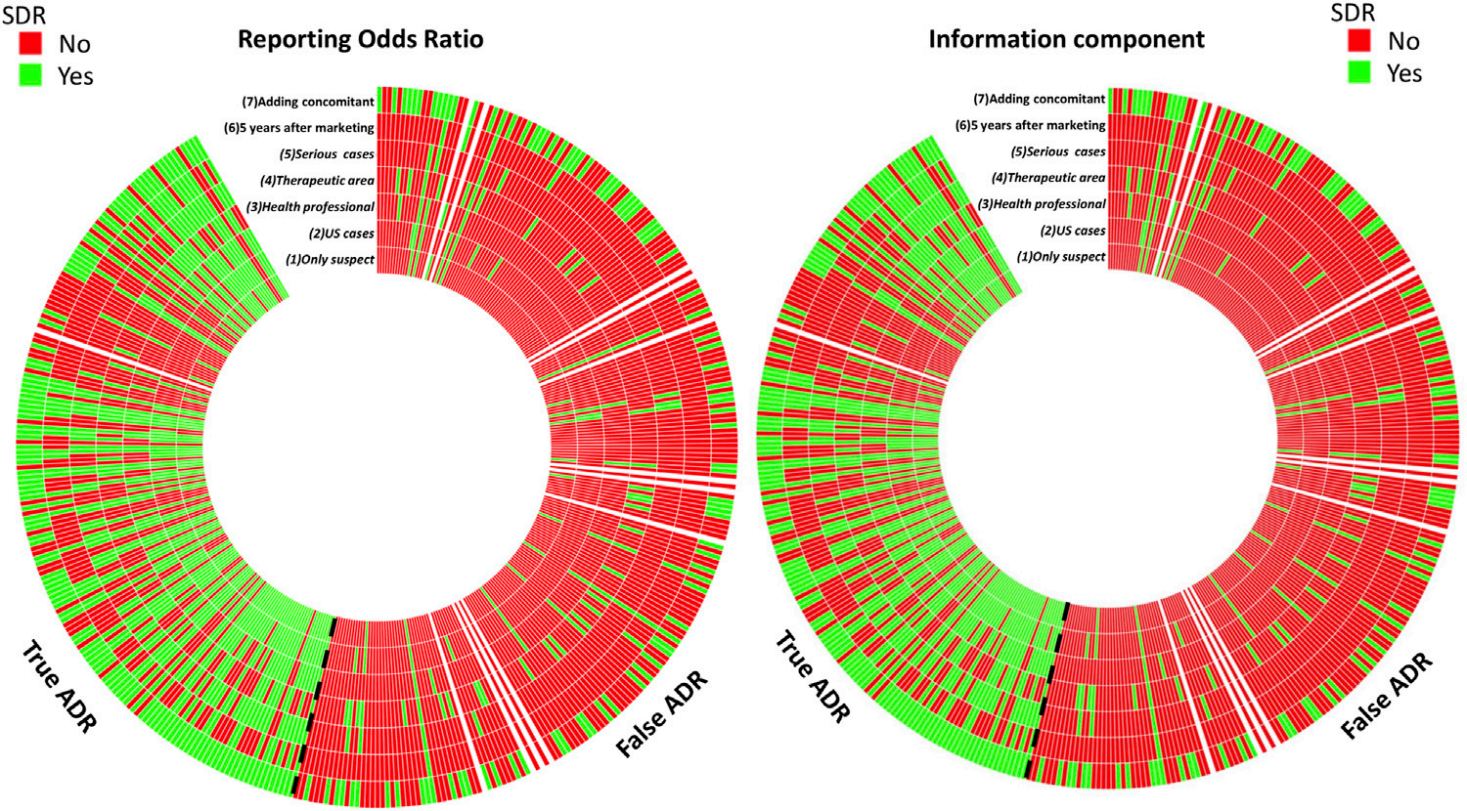
Number of positive and negative signals of disproportionate reporting (SDR) with ROR and IC methods. Each line represents a disproportionality model and each column represents a drug-event pair. A SDR was deemed significant if ROR_LB_ > 1 and *n* > 3 for the ROR method, and IC_LB_ > 0 for the IC method.

The distribution of included cases in each analysis and the results of disproportionality analyses for the seven models are presented in Figure 1 and Table 1 respectively. Over the 399 drug-event pairs, signals could not be examined for four drugs relating to thirteen events in the reference dataset (3.26% of the set) as these drugs were not found in VigiBase. As recommended, ROR values were not computed when the number of exposed cases was lower than 3. This led to lack of ROR value for 4.8–51.9% of the 399 drug-event pairs for the model including concomitant drugs and the model including only ICSRs within 5 years of the drug’s approval, respectively. The ROR_LB_ values varied from 0.01 (gastrointestinal bleeding and neostigmine) to 57.6 (acute liver injury and propylthiouracil) and IC_LB_ values from −17.2 (acute liver injury and miconazole) to 5.1 (acute liver injury and propylthiouracil). Figure 2 presents the SDR generated by the two methods for the 7 models. Overall, we noted high variability in the results and in the number of detected SDRs among the models.

**TABLE 1:** Disproportionality analyses and signal generation results for the lower bound of the 95% confidence intervals of the reporting odds ratio (ROR_LB_) and of the information component (IC_LB_) for the seven selected models. SDR: Signal of disproportionate reporting. Model 1: only suspect cases; Model 2: subgroup by country (United States); Model 3: health professionals only; Model 4: subgroup by therapeutic area; Model 5: serious cases only; Model 6: 5 years after drug approval; Model 7: suspected and concomitant drugs.

	Model 1	Model 2	Model 3	Model 4	Model 5	Model 6	Model 7
ROR_LB_							
N missing (%)	49 (12.3%)	77 (19.3%)	71 (17.8%)	49 (12.3%)	75 (18.8%)	207 (51.9%)	19 (4.8%)
Median	0.48	0.53	0.43	0.61	0.43	0.65	1.16
Q1—Q3	0.15–1.75	0.17–1.97	0.12–1.59	0.28–1.28	0.18–1.62	0.21–2.42	0.59–2.06
Min—Max	0.00–22.39	0.01–57.55	0.00–16.70	0.00–46.57	0.01–22.28	0.00–13.20	0.01–16.74
N positive SDR	122 (33.3%)	120 (30.1%)	107 (26.8%)	117 (29.3%)	104 (26.1%)	73 (18.3%)	212 (53.1%)
N misclassified SDR							
True ADR	59 (35.7%)	60 (36.4%)	71 (43.0%)	86 (52.1%)	73 (44.2%	99 (60%)	40 (24.2%)
False ADR	17 (7.3%)	16 (6.8%)	14 (5.9%)	39 (16.6%)	13 (5.6%))	8 (3.4%)	88 (37.6%)
IC_LB_							
N missing (%)	13 (3.3%)	13 (3.3%)	13 (3.3%)	13 (3.3%)	13 (3.23%)	13 (3.3%)	13 (3.3%)
Median	−1.57	−1.72	−2.14	−0.99	−2.04	−9.99	0.13
Q1—Q3	−3.44–0.46	−4.69–3.67	−4.38–0.16	−3.07–0.13	−4.16–0.03	−10.27–−1.19	−0.94–0.99
Min—Max	−15.45–4.29	−15.70–5.08	−15.40–3.76	−17.24–4.73	−15.90–4.11	−14.50–3.40	−12.70–3.92
N positive SDR	120 (30.1%)	112 (28.1%)	106 (26.6%)	110 (27.6%)	97 (24.3%)	65 (16.3%)	204 (51.1%)
N misclassified SDR							
True ADR	60 (36.4%)	66 (40.0%)	72 (43.6%)	88 (53.3%)	79 (47.8%)	106 (64%)	42 (25.4%)
False ADR	16 (6.8%)	14 (6.0%)	14 (6.0%)	34 (14.5%)	12 (5.1%)	7 (3.0%)	82 (35.0%)

### Evaluation and Comparison of Model Performances

The sensitivity, specificity, AUC and ROC curves corresponding to the seven models are presented in Supplementary Figure S1 and Supplementary Table S2. Overall, in all disproportionality methods, model 4 (subgroup by therapeutic area), model 6 (within 5 years of drug approval) and model 7 (suspected and concomitant drugs) showed limited performances compared to model 1 (including only suspect reports), model 2 (subgroup by country (United States)), model 3 (reports by health professionals) and model 5 (serious cases only). The number of positive and misclassified SDR according to disproportionality methods and models are also presented in Table 1. The number of positive SDR generated ranged from 73 to 212 and 65 to 204 for ROR_LB_ and IC_LB_ respectively. Moreover, the proportion of misclassified SDRs was lower for model 1 and model 2 and systematically higher for true than for false ADR (Table 1).

Of importance, with the ROR method only 37 of the 165 true ADR displayed a SDR with all models, and 125 of the 234 false ADR for negative SDRs. The results were similar with the IC method for which 33 of the 125 true ADRs systematically displayed a signal and 130 of the 234 false ADRs did not (Figure 2).

### Comparison of Disproportionality Results Between True and False ADRs

Median ROR_LB_ values were 1.67 (0.68, 3.91) and 0.39 (0.20, 0.72) for true and false ADRs respectively (*p* < 0.01). Median IC_LB_ values were 0.09 (−1.37, 1.28) and −3.62 (−5.82, −2.22) for true and false ADR respectively (*p* < 0.01). The median number of positive SDR significantly differed between false and true ADR groups, 0 (0, 1) and 5 (1, 6) respectively, in both frequentist and Bayesian methods (Supplementary Table S3).

### Using the Number of Positive SDRs to Improve a Model’s Predictive Capacities

**FIGURE 3:**
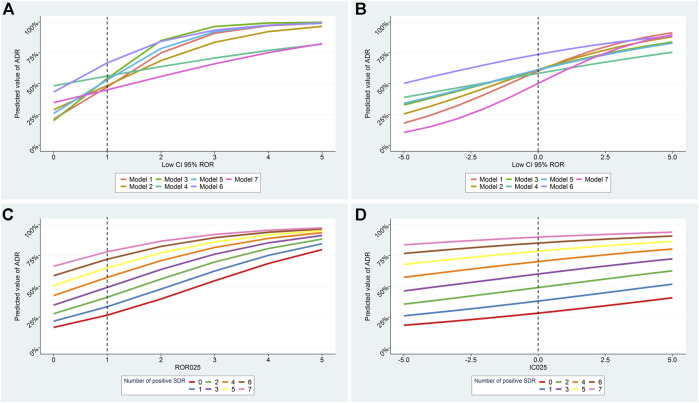
Predicted probability for a signal of disproportionate reporting (SDR) to correspond to a true ADR according to the lower boundary of disproportionality values and to the number of positive SDR. Results of all models according to number of positive SDR for ROR_LB_ and IC_LB_ are presented in **A, B** respectively. Results of model 1 according to the number of positive SDR for ROR_LB_ and IC_LB_ are presented in **C, D**. Model 1: only suspect cases included; Model 2: subgroup by country (United States); Model 3: reporting by health professionals only; Model 4: subgroups by therapeutic area; Model 5: serious cases only; Model 6: within 5 years of drug approval; Model 7: suspected and concomitant drugs.

To investigate whether the number of positive SDR was predictive of a true ADR independently of the disproportionality estimates we performed logistic regression models with and without the inclusion of the number of positive SDR. In all models the number of positive SDR remained independently and significantly predictive of an ADR (Supplementary Table S4). The lower boundary of ROR and IC were no longer significant after adjustment on the number of positive SDR in 5 of the models. The predictive capacities of all methods and models are plotted in Figure 3, Supplementary Figures S2, S3. The number of positive SDR strongly impacted the predictive capacities of all models notably for lower boundary values close to the threshold of signal detection for ROR and IC.

## Discussion

To our knowledge, this is the first study assessing the variability of disproportionality analyses using several models and methods. We showed that the number of positive SDR in a standardized set of 7 models can serve as a significant predictor of a true ADR. Importantly, this property of the number of positive SDR was independent of disproportionality values and could therefore be used to further investigate the plausibility of a signal.

Our study also underlines the necessity of reporting multiple secondary or sensitivity analyses when studying a drug-event association through disproportionality analyses. Indeed, almost 60% of all drug-event pairs displayed both positive and negative SDR regardless of their truthfulness. This finding highlights the potential risk of reporting bias in disproportionality analyses studies, especially since in this study we used a single threshold to define a signal, whereas this is not the case in all studies (Candore et al., 2015). This risk is particularly important in the pharmacovigilance field where the data are openly accessible to researchers, no protocols are pre-registered and no gold standard methods have yet been established (Wisniewski et al., 2016).

Performing a set of analyses, rather than only one, is all the more important as their variability seems to be associated with the probability of being a true finding. The impact of this variability is particularly important when disproportionality values are close to the threshold of signal detection (ROR_LB_ > 1 or IC_LB_ > 0), for which the probability of a result being true may vary from 30 to 80% depending of the number of positive SDR. Unexpectedly, the disproportionality values were no longer significant when adjusted on the number of positive SDR in 5 of the 7 models for both methods, stressing the relevance of this metric. In this study, we pre-defined a set of analyses for which the results are almost exclusively impacted by the random variability in ADR reporting rates, unrelated to ADRs or patients characteristics. However, we cannot exclude that real differences in the ADR relative risk may exist for some individual drugs according, for example, to the reporting country (Sandberg et al., 2020). Further work is thus needed to select the best set of analyses.

Although this study did not primarily aim to compare the performance of disproportionality analysis models and methods, it supplements the knowledge on this topic. Importantly, we observed very similar results between frequentist and Bayesian methods, in accordance with some previous studies (Candore et al., 2015; Pham et al., 2019). This might indicate that our results could be generalized to other methods. In addition, we found comparable performance between four models: all suspect reports included, limiting reports from a given country, including only reports by health professionals, and including only serious cases, with a lower proportion of misclassified SDR when using the first two methods. In contrast, models restricting comparison to a given therapeutic area, including only cases reported within 5 years of drug approval, or including concomitant reports, were inferior whatever the method. An earlier study conducted by Seabroke et al. likewise showed an appreciable benefit associated with subgrouping by reporter qualification or by country of origin, but little advantage using severity (Seabroke et al., 2016). This discrepancy might be explained by a slightly different definition of a positive signal (subgroup analyses were examined in each strata and a SDR was found significant if the criteria were met in any of the strata). In 2013 Harpaz et al. compared the performances of several disproportionality methods using the FDA adverse event reporting database using the same reference set (Harpaz et al., 2013). They also found a better specificity than sensitivity of the reported odds ratio but the overall performances were inferior to those in our study. That may be due to the greater number of cases included in our study (21 million vs. 5 million) and broader definitions of outcomes, consistent with other studies that found higher performances using larger databases and similar event definitions (Reich et al., 2013; Caster et al., 2020).

Our study had some limitations. The reference dataset used here is one of the largest to date that incorporates both SPCs and evidence from literature reviews. However, Hauben et al. suggested that a small part of false drug-event pairs may be misclassified, which could impact the performances of disproportionality analyses (Hauben et al., 2016). Nonetheless, the comparisons between models and methods are unlikely to have been affected by this bias. Moreover, to standardize the models we had to make choices (e.g. 5 year after drug approval for model 6, United States country for model 2 or defining the therapeutic area by the ATC level 3 in model 4). These standardized definitions may not be relevant for some drug-event pairs (e.g. for drugs not commercialized in the United States or belonging to an heterogenous ATC class) and have to be further adapted to the nature of the studied drug and ADR. The findings of our study may not be generalizable to other databases due to differences related to the database background and the medical products covered. Nevertheless, several studies have highlighted the similitudes and overlaps between SDR found from pharmaceutical company databases and international pharmacovigilance databases (Candore et al., 2015; Vogel et al., 2020). Finally, while it can be assumed that the results are applicable to other drug events pairs, the predicted probabilities calculated in this study are not extrapolable.

## Conclusion

To conclude, this study shows the wide variability of disproportionality analysis results depending on the method and model specifications, thus opening the door for selective reporting of results. We therefore advocate for the pre-registration of protocols and the presentation of a set of secondary and sensitivity analyses instead of a unique result to limit reporting bias and because variability in the results may reflect the likelihood of a signal being a true adverse drug reaction.

## Data Availability

The datasets presented in this study can be found in online repositories. The names of the repository/repositories and accession number(s) can be found below: The protocol, data and R codes underlying this article could be found on Open Science Framework (osf.io/a7j3z/).
