# Supplementary material for: Leveraging the Variability of Pharmacovigilance Disproportionality Analyses to Improve Signal Detection Performances
Source: Front Pharmacol. 2021 May 28;12:668765. doi: 10.3389/fphar.2021.668765 (PMC8193489; doi:10.3389/fphar.2021.668765)

**Leveraging the variability of pharmacovigilance disproportionality analyses to improve signal detection performances.**

Supplementary material

Table of content

[Table S1. MedDra terms used for events definition in VigiBase. 2](#_Toc63438997)

[Table S2. Sensibility, specificity and AUC of the lower boundary of 95% confidence intervals of the reporting odds ratio (ROR_025_) at a threshold of 1 and of the lower boundary of 95% confidence intervals for the information component (IC_025_) at a threshold of 0 ,according to the seven models. 3](#_Toc63438998)

[Table S3. Median disproportionality values and median number of positive SDR between true and false ADR groups. Comparison of distributions through Mann-Whitney-Wilcoxon tests. 3](#_Toc63438999)

[Figure S1. ROC curve of the seven models for ROR_LB_ and IC_LB._ 4](#_Toc63439000)

[Table S4. Performance of logistic regression models for prediction of ADR with and without the number of positive signals for each method and model. 5](#_Toc63439001)

[Figure S2. Predictive capacities of all model combined with the number of positive SDR with ROR_LB_ 6](#_Toc63439002)

[Figure S3. Predictive capacities of all model combined with the number of positive SDR with IC_LB_ 7](#_Toc63439003)

# Table S1. MedDra terms used for events definition in VigiBase.

| Adverse drug reactions | MedDra terms |
| --- | --- |
| Acute renal failure | Acute renal failure (SMQ narrow) |
| Myocardial infarction | Myocardial infarction (SMQ narrow) |
| Acute liver injury | Acute liver injury (PT)  Acute hepatic failure (PT)  Coma hepatic (PT)  Hepatic encephalopathy (PT)  Hepatic failure (PT)  Hepatic necrosis (PT)  Hepatitis fulminant (PT)  Liver transplant (PT) |
| Gastrointestinal bleeding | Anal haemorrhage (PT)  Anal ulcer haemorrhage (PT)  Anastomotic ulcer haemorrhage (PT)  Duodenal ulcer haemorrhage (PT)  Duodenitis haemorrhagic (PT)  Gastric haemorrhage (PT)  Gastric ulcer haemorrhage (PT)  Gastric ulcer haemorrhage, obstructive (PT)  Gastritis haemorrhagic (PT)  Gastrointestinal haemorrhage (PT)  Gastrointestinal ulcer haemorrhage (PT)  Gastroduodenal haemorrhage (PT)  Haematemesis (PT)  Haematochezia (PT)  Haemorrhagic erosive gastritis (PT)  Intestinal haemorrhage (PT)  Intra-abdominal haemorrhage (PT)  Large intestinal haemorrhage (PT)  Large intestinal ulcer haemorrhage (PT)  Lower gastrointestinal haemorrhage (PT)  Melaena (PT)  Mesenteric haemorrhage (PT)  Oesophageal haemorrhage (PT)  Oesophageal ulcer haemorrhage (PT)  Oesophagitis haemorrhagic (PT)  Peptic ulcer haemorrhage (PT)  Rectal haemorrhage (PT)  Small intestinal haemorrhage (PT)  Small intestinal ulcer haemorrhage (PT)  Upper gastrointestinal haemorrhage (PT) |

SMQ: Standardised MedDRA Queries (groupings of MedDRA terms); PT: preferred term

# Table S2. Sensibility, specificity and AUC of the lower boundary of 95% confidence intervals of the reporting odds ratio (ROR_025_) at a threshold of 1 and of the lower boundary of 95% confidence intervals for the information component (IC_025_) at a threshold of 0 ,according to the seven models.

|  | **ROR_LB_** | | | **IC_LB_** | | |
| --- | --- | --- | --- | --- | --- | --- |
| **Model** | **specificity** | **sensitivity** | **AUC** | **specificity** | **sensitivity** | **AUC** |
| **1** | 89.58 | 67.52 | 86.76 | 92.79 | 63.41 | 85.28 |
| **2** | 89.88 | 67.53 | 85.90 | 93.69 | 59.76 | 82.86 |
| **3** | 90.91 | 61.59 | 85.48 | 93.69 | 56.10 | 82.86 |
| **4** | 76.56 | 49.68 | 70.61 | 84.68 | 46.95 | 73.94 |
| **5** | 90.80 | 62.00 | 87.16 | 94.59 | 51.83 | 83.17 |
| **6** | 85.29 | 56.45 | 78.85 | 96.85 | 35.37 | 78.47 |
| **7** | 59.45 | 76.07 | 75.28 | 62.61 | 74.39 | 75.32 |

# Table S3. Median disproportionality values and median number of positive SDR between true and false ADR groups. Comparison of distributions through Mann-Whitney-Wilcoxon tests.

|  | Group | N | Median | p-value |
| --- | --- | --- | --- | --- |
| ROR_LB_ values | False | 217 | 0.39 (0.20, 0.72) | <0.001 |
|  | True | 163 | 1.67 (0.68, 3.91) |  |
| Number of positive SDR | False | 193 | 0 (0,1) | <0.001 |
|  | True | 164 | 5 (1,6) |  |
| IC_LB_ values | False | 222 | -3.62 (-5.82, -2.22) | <0.001 |
|  | True | 164 | 0.09 (-1.37, 1.28) |  |
| Number of positive SDR | False | 222 | 0 (0,1) | <0.001 |
|  | True | 164 | 5 (1,6) |  |

# Figure S1. ROC curve of the seven models for ROR_LB_ and IC_LB._


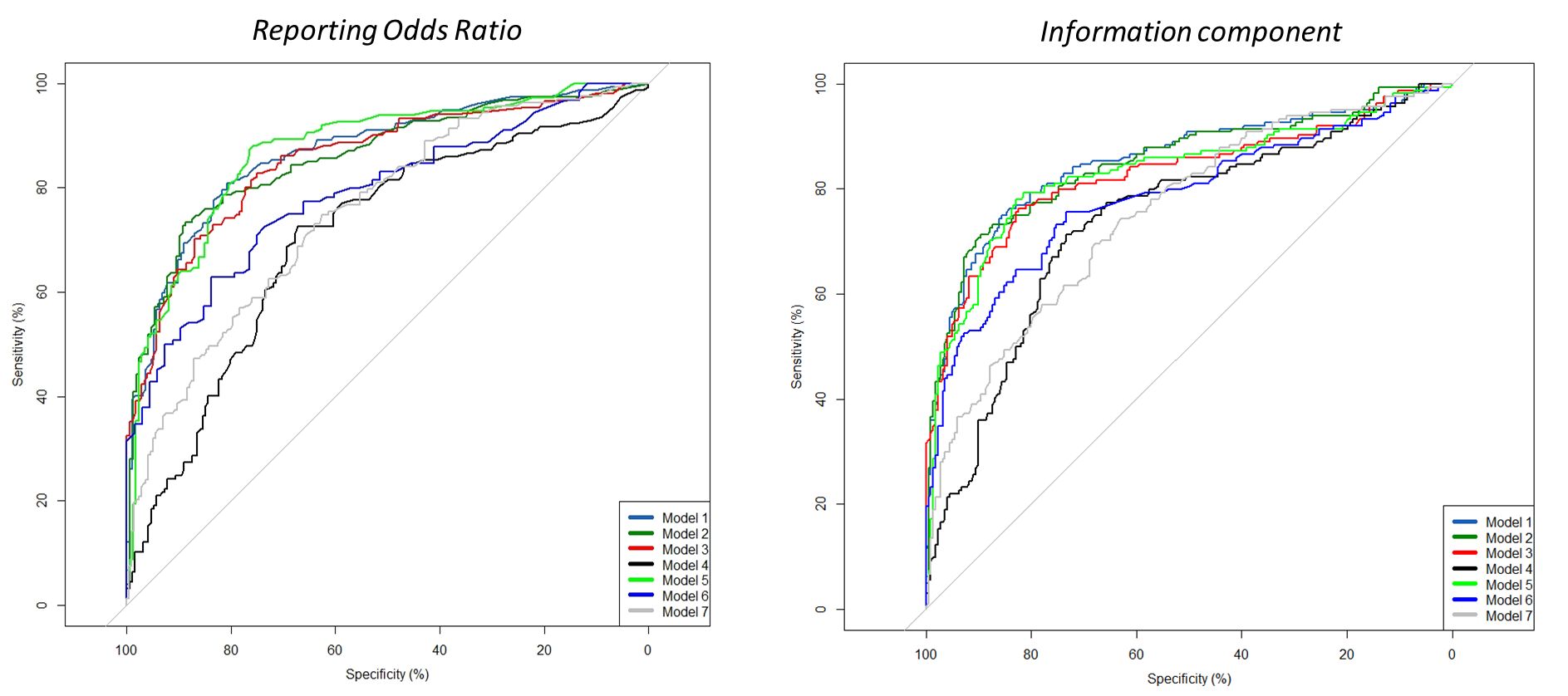


# Table S4. Performance of logistic regression models for prediction of ADR with and without the number of positive signals for each method and model.

|  |  |  | **Reporting Odds Ratio** | | | | | | **Information Component** | | | | | |
| --- | --- | --- | --- | --- | --- | --- | --- | --- | --- | --- | --- | --- | --- | --- |
|  | Predictor |  | Multivariable-adjusted OR without  number of SDR | p-value | AIC | Multivariable-adjusted OR with  number of SDR | value | AIC | Multivariable-adjusted OR without  number of SDR | p-value | AIC | Multivariable-adjusted OR with  number of SDR | value | AIC |
| model 1 | Low CI |  | 3.34 (2.37, 4.97) | **<0.001** | 337.4 | 1.71 (1.13, 3.04) | **0.035** | 332.0 | 1.47 (1.32, 1.67) | **<0.001** | 403.2 | 1.12 (1.001, 1.27) | 0.053 | 375.4 |
|  | Number of drug-event pairs |  | 1.00 (0.99, 1.00) | 0.220 |  | 1.00 (0.99, 1.00) | 0.266 |  | 1.00 (1.00, 1.00) | **0.043** |  | 1.00 (1.00, 1.00) | 0.209 |  |
|  | Number of positive SDR |  |  |  |  | 1.38 (1.10, 1.71) | **0.005** |  |  |  |  | 1.56 (1.33, 1.85) | **<0.001** |  |
| model 2 | Low CI |  | 2.33 (1.76, 3.26) | **<0.001** | 328.3 | 1.34 (1.05, 1.91) | 0.056 | 314.2 | 1.36 (1.25, 1.49) | **<0.001** | 399.5 | 1.12 (1.04, 1.25) | **0.009** | 371.9 |
|  | Number of drug-event pairs |  | 1.00 (1.00, 1.00) | 0.076 |  | 1.00 (1.00, 1.00) | 0.201 |  | 1.00 (1.00, 1.00) | **0.032** |  | 1.00 (1.00, 1.00) | 0.225 |  |
|  | Number of positive SDR |  |  |  |  | 1.45 (1.21, 1.75) | **<0.001** |  |  |  |  | 1.52 (1.30, 1.78) | **<0.001** |  |
| model 3 | Low CI |  | 4.77 (3.08, 7.90) | **<0.001** | 313.6 | 2.07 (1.23, 3.92) | **0.015** | 304.2 | 1.27 (1.17, 1.38) | **<0.001** | 426.4 | 1.04 (0.96, 1.14) | 0.312 | 378.8 |
|  | Number of drug-event pairs |  | 1.00 (1.00, 1.00) | 0.406 |  | 1.00 (1.00, 1.00) | 0.388 |  | 1.00 (1.00, 1.00) | **0.014** |  | 1.00 (1.00, 1.00) | 0.198 |  |
|  | Number of positive SDR |  |  |  |  | 1.41 (1.16, 1.72) | **0.001** |  |  |  |  | 1.66 (1.43, 1.95) | **<0.001** |  |
| model 4 | Low CI |  | 1.38 (1.16, 1.70) | **0.001** | 416.1 | 0.97 (0.89, 1.09) | 0.533 | 339.4 | 1.17 (1.08, 1.28) | **<0.001** | 454.7 | 0.97 (0.90, 1.05) | 0.437 | 379.3 |
|  | Number of drug-event pairs |  | 1.00 (1.00, 1.00) | **<0.001** |  | 1.00 (1.00, 1.00) | 0.148 |  | 1.00 (1.00, 1.00) | **<0.001** |  | 1.00 (1.00, 1.00) | 0.160 |  |
|  | Number of positive SDR |  |  |  |  | 1.77 (1.55, 2.04) | **<0.001** |  |  |  |  | 1.79 (1.55, 2.09) | **<0.001** |  |
| model 5 | Low CI |  | 3.24 (2.28, 4.87) | **<0.001** | 332.5 | 1.28 (0.95, 2.02) | 0.223 | 305.9 | 1.25 (1.16, 1.63) | **<0.001** | 430.7 | 1.05 (0.97, 1.14) | 0.250 | 378.5 |
|  | Number of drug-event pairs |  | 1.00 (1.00, 1.00) | 0.146 |  | 1.00 (1.00, 1.00) | 0.234 |  | 1.00 (1.00, 1.00) | **0.009** |  | 1.00 (1.00, 1.00) | 0.201 |  |
|  | Number of positive SDR |  |  |  |  | 1.63 (1.36, 1.96) | **<0.001** |  |  |  |  | 1.66 (1.44, 1.94) | **<0.001** |  |
| model 6 | Low CI |  | 2.66 (1.78, 4.48) | **<0.001** | 202.0 | 1.41 (0.99, 2.36) | 0.122 | 186.1 | 1.23 (1.17, 1.29) | **<0.001** | 407.7 | 1.14 (1.07, 1.21) | **0.000** | 362.3 |
|  | Number of drug-event pairs |  | 1.00 (1.00, 1.00) | 0.222 |  | 1.00 (1.00, 1.00) | 0.722 |  | 1.00 (1.00, 1.00) | **0.011** |  | 1.00 (1.00, 1.00) | 0.382 |  |
|  | Number of positive SDR |  |  |  |  | 1.49 (1.24, 1.84) | **<0.001** |  |  |  |  | 1.57 (1.37, 1.81) | **<0.001** |  |
| model 7 | Low CI |  | 1.55 (1.30, 1.89) | **<0.001** | 433.5 | 0.94 (0.80, 1.13) | 0.466 | 369.9 | 1.54 (1.31, 1.85) | **<0.001** | 440.3 | 0.98 (0.86, 1.14) | 0.762 | 379.8 |
|  | Number of drug-event pairs |  | 1.00 (1.00, 1.00) | **0.001** |  | 1.00 (1.00, 1.00) | 0.141 |  | 1.00 (1.00, 1.00) | **0.001** |  | 1.00 (1.00, 1.00) | 0.164 |  |
|  | Number of positive SDR |  |  |  |  | 1.79 (1.54, 2.11) | **<0.001** |  |  |  |  | 1.76 (1.52, 2.06) | **<0.001** |  |

# Figure S2. Predictive capacities of all model combined with the number of positive SDR with ROR_LB_


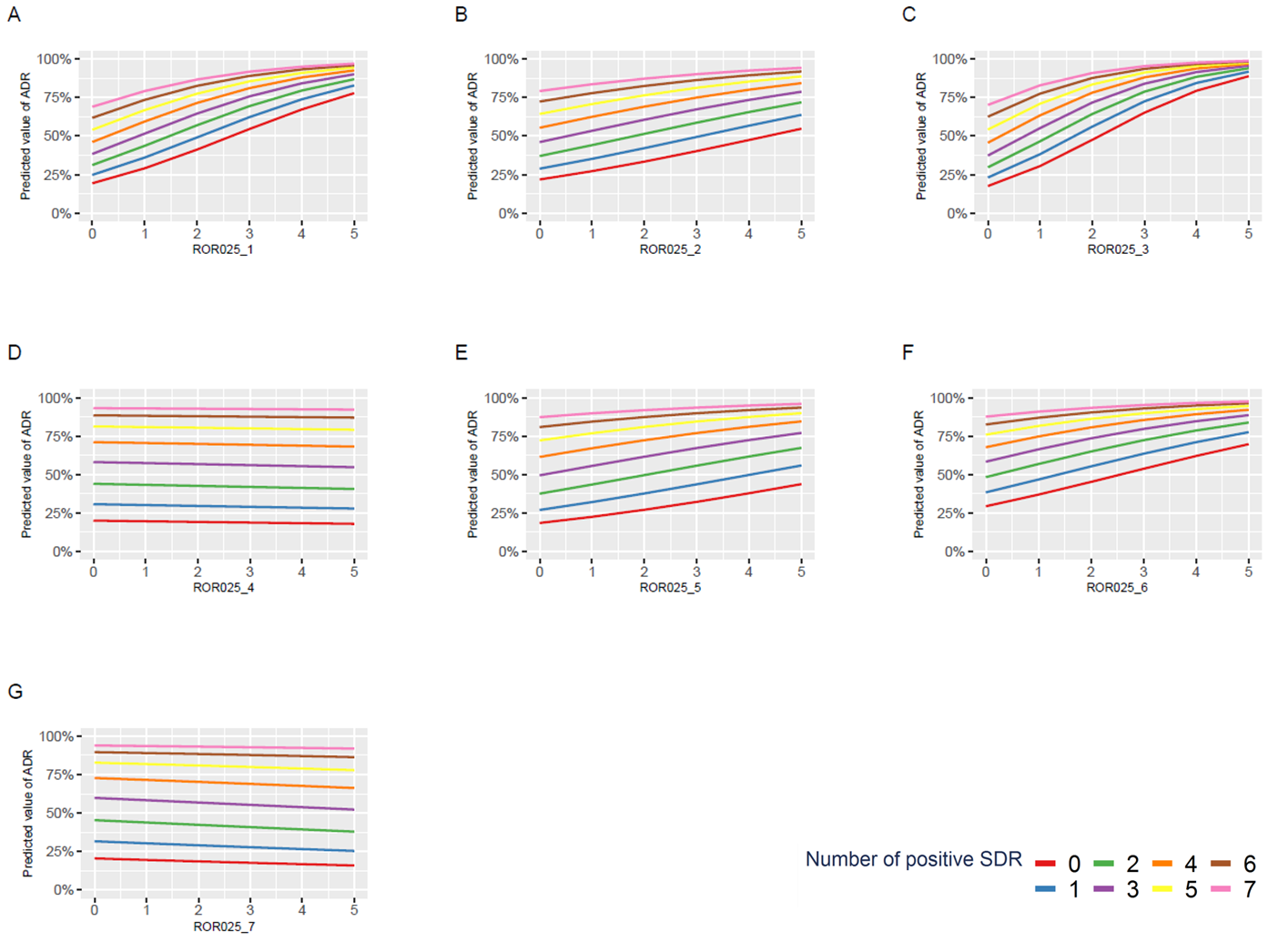


# Figure S3. Predictive capacities of all model combined with the number of positive SDR with IC_LB_


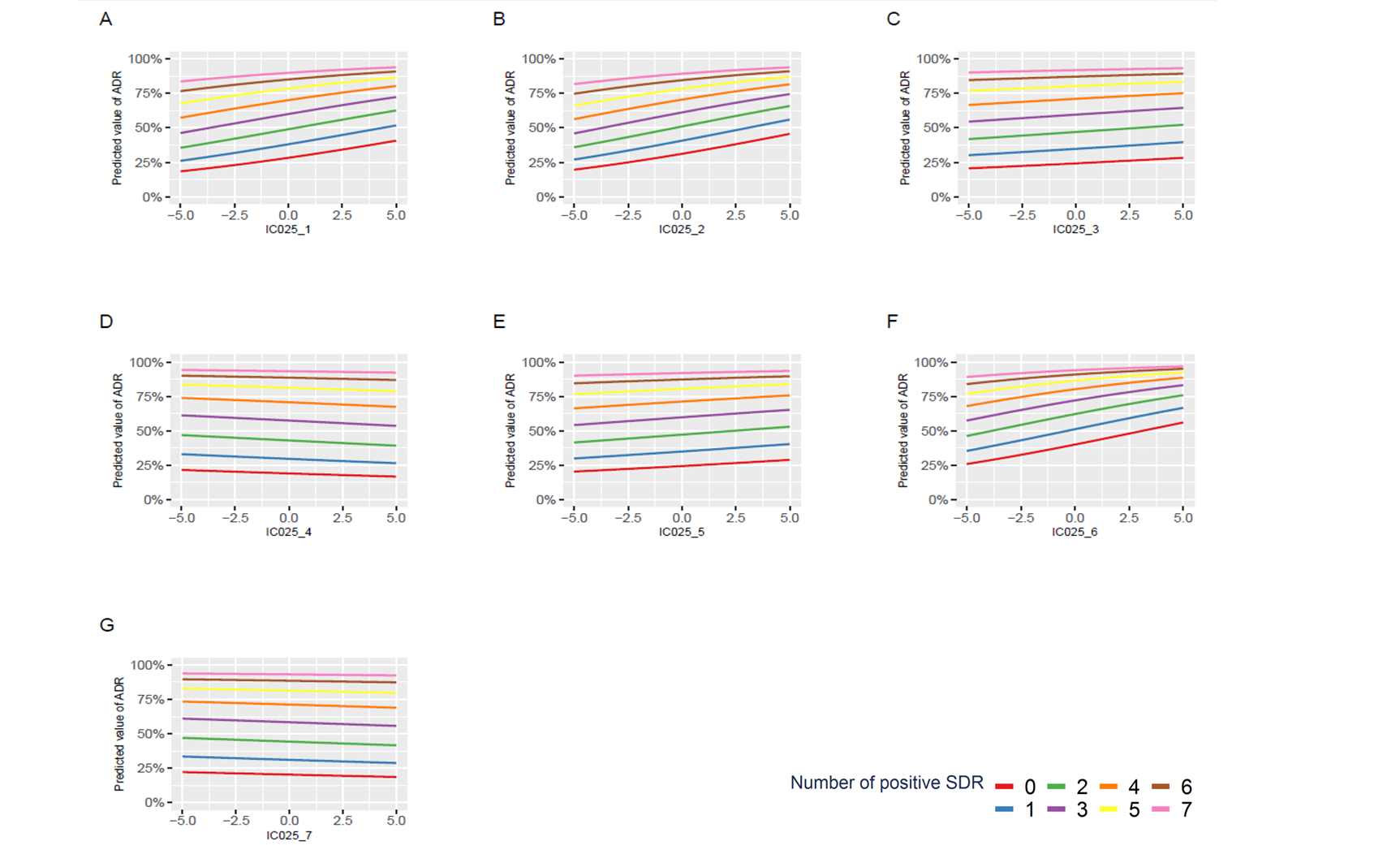

Supplement: Supplementary file 1 [file DataSheet1.DOCX]
